# Ablation of the Locus Coeruleus Increases Oxidative Stress in Tg-2576 Transgenic but Not Wild-Type Mice

**DOI:** 10.4061/2010/864625

**Published:** 2010-10-11

**Authors:** Orest Hurko, Kurt Boudonck, Cathleen Gonzales, Zoe A. Hughes, J. Steve Jacobsen, Peter H. Reinhart, Daniel Crowther

**Affiliations:** ^1^Biologics Consulting Group, Inc., 400 N. Washington Street, Suite 100, Alexandria, VA 22314, USA; ^2^Department of Medicine, Nursing, and Dentistry, University of Dundee, Nethergate, Dundee DD1 4HN, UK; ^3^Metabolon Inc., 800 Capitola Drive, Suite 1, Durham, NC 27713-4385, USA; ^4^Bayer Cropscience, 3500 Paramount Parkway Morrisville, NC 27560-7218, USA; ^5^Pfizer, 558 Eastern Point Road Groton, CT 06340-5196, USA; ^6^Proteostasis Therapeutics, 200 Technology Square, Suite 402, Boston, MA 02139, USA; ^7^Department of Neuroscience, Duke University, Durham, NC 27708, USA; ^8^Pfizer, Translational Medicine Research Collaboration, Dundee DD1 9SY, UK

## Abstract

Mice transgenic for production of excessive or mutant forms of beta-amyloid differ from patients with Alzheimer's disease in the degree of inflammation, oxidative damage, and alteration of intermediary metabolism, as well as the paucity or absence of neuronal atrophy and cognitive impairment. Previous observers have suggested that differences in inflammatory response reflect a discrepancy in the state of the locus coeruleus (LC), loss of which is an early change in Alzheimer's disease but which is preserved in the transgenic mice. In this paper, we extend these observations by examining the effects of the LC on markers of oxidative stress and intermediary metabolism. We compare four groups: wild-type or Tg2576 A*β* transgenic mice injected with DSP4 or vehicle. Of greatest interest were metabolites different between ablated and intact transgenics, but not between ablated and intact wild-type animals. The Tg2576_DSP4 mice were distinguished from the other three groups by oxidative stress and altered energy metabolism. These observations provide further support for the hypothesis that Tg2576 A*β* transgenic mice with this ablation may be a more congruent model of Alzheimer's disease than are transgenics with an intact LC.

## 1. Introduction

The degree of congruence between animal models and human disease is likely to be a measure of how faithfully preclinical compound validation will translate into efficacy in the clinic. Both academic and industrial laboratories have made major investments in investigations of putative disease-modifying treatments for Alzheimer's disease, relying on various mouse models transgenic for structural variants or elevated quantities of beta-amyloid. Among them are the Tg2576 mice which express the Swedish mutation of APP (APP_K670N,  M671L_) [1], which is the focus of this study. This mutation causes increases in secreted A*β*1-40 and A*β*1-42 [2, 3]. Although this and other transgenic models reproducibly lead to pathological accumulations of beta-amyloid, they poorly mimic several key pathologic features of human Alzheimer's disease: gross brain atrophy and profound dementia [4]. This incongruence may compromise their utility for validation for putative disease-modifying treatments, particularly those directed not on amyloid accumulation, but on downstream pathophysiology. 

Several lines of evidence indicate that features other than amyloid may also play critical roles in the pathological cascade leading to the severe brain atrophy and progressive dementia that are the ultimate clinical concern in nonmendelian Senile Dementia of the Alzheimer Type (SDAT), which in the last decade has come to be called Alzheimer disease (AD), as it will be referred to in this paper. The most compelling come from two independent prospective observational studies: the Religious Orders study in Kentucky and the adjacent Midwest [5, 6] and the similar, MRC Cognitive Function and Ageing Study (CFAS) [7]. In both studies, subjects were assessed periodically for cognitive ability and after death, their brains were examined. In both studies, the relationship between amyloid burden and cognitive impairment was complex. There were many individuals whose total burden of amyloid pathology was incongruent with their normal cognitive status, even when all pathologies were considered in a multivariable model of dementia risk factors. These observations suggested that a congruent rodent model of AD also requires more than just alterations of amyloid.

Although not understood completely, two biochemical features of AD are oxidative damage [8–22] and inflammation [23]. Although these are prominent in AD, they are variable in amyloid transgenic mice [24–29]. Another change in human AD—but also absent or minor in transgenic rodents—is early degeneration of the locus coeruleus (LC) [30–41]. Work by Heneka et al. [42–45] suggested that there may be a link between loss of the noradrenergic LC and inflammatory changes in AD. The purpose of the experiments described in this paper was to determine if there is a similar link with oxidative stress and other metabolic changes.

 The LC is a midline brainstem nucleus that is the origin of a widely projecting noradrenergic system, the major site of norepinephrine synthesis in the brain that projects widely to the neocortex and the hippocampus with terminals on neurons, glia, and blood vessels [46]. Loss of noradrenergic cells of the LC is an early event in human AD, comparable to the more widely recognized loss of cells from the cholinergic midline projection system originating in the substantia innominata [40].

 Heneka et al. [42] injected wild-type adult rats with DSP4 [47, 48] to eliminate noradrenergic LC neurons and then challenged with intracortical injections of A*β*. The ablated rats showed greater induction of inflammatory nitric oxide synthase, interleukin 1*β*, and IL-6 expression in response to injection with A*β* than did control animals in which the LC had been left intact. This demonstrated that LC loss in the rats augments inflammatory responses to A*β*. In a followup study, APP23 transgenic mice overexpressing A*β* were injected with DSP4 to selectively destroy noradrenergic neurons in the LC as a model for Alzheimer's disease [45], with similar increases in inflammatory response. These observations invited our hypothesis that LC loss in AD might be permissive not only for increased inflammation but also for potentiation of oxidative stress. 

The specific goals of the current study were to determine if the same ablative procedure of the LC with DSP4 in Tg2576 mice would significantly alter other metabolic pathways that are not affected by transgenesis for amyloid in mice with an intact LC and to determine if such alterations are typical of human AD. Furthermore, we were interested in determining only those metabolic changes resulting from this ablation that were also dependent on transgenesis for amyloid and were not simply nonspecific consequences of treatment with DSP4 in control wild-type mice.

To ensure a comprehensive unbiased survey, we chose to perform a metabolomic analysis. Metabolomics has attracted increasing interest in the field of disease research, since it has proven to be a fast and reproducible method directly reflecting biological events. Metabolomics involves the determination of changes in the levels of endogenous or exogenous metabolites in biological samples, in response to a perturbation such as disease, drug, or toxin. In the present study, we used a combination of gas chromatography/mass spectrometry (GC/MS) and liquid chromatography/mass spectrometry (LC/MS) to monitor the biochemical changes associated with a transgenic murine model system for Alzheimer's disease. This global metabolite profiling allows the measurement of low molecular weight biochemicals including lipids, sugars, nucleotides, organic acids, amino acids, vitamins, cofactors, small peptides, and xenobiotics.

## 2. Materials and Methods

### 2.1. Neurochemical Analysis of Brain Tissue

DSP4 (50 mg/kg in PBS, *N* = 5) or PBS (vehicle, *N* = 5) was administered intraperitoneally to C57BL/6SJL mice on days 1 and 7. Mice were sacrificed 7 days following the second injection (day 14). Mice were euthanized by exposure to CO_2_ and the brains removed. Frontal cortex tissue was dissected on ice and frozen on dry ice for subsequent analysis of monoamine concentration. Frozen tissue samples were weighed and perchloric acid (0.4 M) added at 10 *μ*L/mg tissue. Samples were made homogeneous using a sonicating probe. Samples were then centrifuged at 15 000 rpm for 20 min and the supernatant removed. The supernatant was then centrifuged in a filtered tube at 3000 rpm for 5 min. This supernatant was then diluted 1 in 100 for measurement of dopamine and 1 in 10 for measurement of DOPAC and HVA. Supernatant was analyzed for neurochemical content using HPLC-ECD. Chromatographic separations were performed using reverse phase HPLC (C18 ODS3 column, 150 × 3.0 mm, Metachem, Torrance, CA, USA). The mobile phase comprised: 0.15 M NaH_2_PO_4_, 0.25 mM EDTA, 1.75 mM 1-octane sulphonic acid, 2% isopropanol and 4% methanol, pH = 4.6 and delivered at 0.5 mL/min at 30°C. Dopamine and metabolites were detected using an electrochemical amperometric detector (Decade, Antec-Leyden, NL) with a working electrode set at +650 mV versus Ag/AgCl reference electrode.

### 2.2. Preparation of Mice for Spectroscopic Analyses

A total of 120 samples from mice were used in this study, comprising 4 sets of 23 week old animals (*n* = 10/group): either wild-type or Tg2576 A*β* transgenics which express the Swedish mutation (APP_K670N,  M671L_) of the amyloid protein precursor (APP) [1]. Each 23 week-old animal was injected intraperitoneally on day 1, (5 weeks prior to harvest) with either vehicle (PBS) or DSP4 (N-(2-chloroethyl)-N-ethyl-2-bromobenzylamine, Sigma C-8417), a selective neurotoxin for noradrenergic neurons [47, 48] dissolved in PBS. Each animal was injected intraperitoneally with a volume of 5 mL/kg. An identical injection was given to each animal 7 days later (on day 8) when animals were 24 weeks old. Four weeks after the second injection, the mice were anesthetized with 3% isoflurane and plasma, CSF, and brains (40 samples each) were harvested for metabolite profiling, as described below.

### 2.3. Sample Collection for Spectroscopic Analyses

Approximately 7 microliters of CSF was collected from each mouse *via* the cisterna magna and visually inspected for the presence of blood. Any sample that was contaminated with blood was removed from the study. The collected CSF was immediately frozen on dry ice. For the collection of plasma, the chest cavity was opened and approximately 0.3 mL of blood was collected via cardiac puncture into EDTA tubes and the mice were euthanized by exsanguination. The blood samples were centrifuged to separate plasma. The plasma was collected and immediately frozen on dry ice. Brains were extracted from the skull and frozen on dry ice. For these brain samples, the cerebellum was left intact. 

The plasma (100 *μ*L), CSF (approximately 7 *μ*L) and brain samples (half brain, 150 mg) were extracted according to Metabolon's standard protocol, which uses a series of proprietary solvent extractions designed to remove protein and dislodge any small molecules bound to protein or physically trapped in the precipitated protein matrix. Each solvent extraction step was performed by shaking for two minutes in the presence of glass beads using a Glen Mills Genogrinder 2000. After each extraction the sample was centrifuged and the supernatant removed using the MicroLab STAR robotics system, followed by re-extraction of the pellet in subsequent steps. The multiple extract supernatants were pooled and then split into equal parts for analysis on the GC/MS and LC/MS platforms. Further details on Metabolon's extraction procedures and metabolic profiling platform can be found in Boudonck et al. [49, 50] and Evans et al. [51].

### 2.4. Data Collection and Quality Control

The data were collected over two platform day runs each for plasma, CSF, and brain matrix. Each day consisted of 19 or 20 study samples, with samples from the different treatment groups equally distributed among the days, then randomized. Two brain samples did not pass QC inspection and were omitted from the study. Approximately 30% of process samples were dedicated to QC. In addition to the study samples, pooled samples of homogenized plasma, CSF, and brain tissue were extracted four independent times per day. These samples (CMTRX) served as technical replicates throughout the data set to assess process variability. Also, 100 *μ*L of water was extracted five independent times per day to serve as process blanks. Every sample analyzed was spiked with standards to monitor and evaluate instrument and extraction performance [49].

### 2.5. Data Normalization

Raw area counts for each metabolite in each sample were normalized to correct for variation resulting from instrument interday tuning differences [49]. For each metabolite, the area counts were divided by its median value for each run day, therefore setting the medians equal to 1 for each day's run. This correctly preserves all of the variation between the samples yet allows metabolites of widely different raw peak areas to be compared directly on a similar graphical scale. Missing values were assumed to result from areas falling below the limits of detection. For each metabolite, the missing values were imputed with the observed minimum after the normalization step. Additional normalization steps were added to correct for the volume differences between samples in the different matrices. For plasma, several samples had volumes lower than 100 *μ*L and to those samples a correction factor was applied. For CSF, sample volumes varied between 5 and 10 *μ*L. Therefore, a correction factor was applied to each sample based on the total ion count for all metabolites present in the sample. For brain samples a correction factor based on the total ion count for all metabolites present in the sample was applied as well, as in the case for CSF.

### 2.6. Statistical Analysis

Individual contrast analyses were performed between various groups of the study. The following comparisons were made: Tg2576_DSP4 versus wild type_DSP4, Tg2576_vehicle versus wild-type_vehicle, Tg2576_DSP4 versus Tg2576_vehicle, and wild-type_DSP4 versus wild-type_vehicle. In addition, a two-way (factorial) ANOVA analysis was performed to test whether the mean difference between genotype depends on the drug (or vice-versa). Data were log-transformed for all tests. A conservative *P*-value cutoff of  .01 was used for this study. To account for multiple testing, false discovery rates (FDRs) were computed for each comparison [52]. The FDRs were estimated using the *Q* value method [53]. The *Q* value is a measure of each metabolite's significance that takes into account the hundreds of comparisons that we are making simultaneously in our analysis. The *Q* value, which is an extension of the FDR, is similar to the more familiar *P* value, which is a measure of the false positive rate. We use it in our univariate analyses to minimize the occurrence of potentially misleading false positives that would be otherwise expected with the performance of this many simultaneous comparisons.

### 2.7. Principal Component Analysis (PCA) and Partial Least Squares-Discriminant Analysis (PLS-DA)

Principal components analysis (PCA) is a dimension reduction method that uses a linear transformation of a sample of points to exhibit the properties of the sample most clearly along the coordinate axes [54]. This analysis is now routinely used in the analysis of genome scale data sets, for example, [55]. The principal components (PCs) are displayed as a set of “scores”, which highlight clustering or outliers, and a set of “loadings”, which highlight the influence of input variables on *t*. PCA and PLS-DA analysis were completed in SIMCA P v12.0 (Umetrics AB) on a desktop PC running Windows XP Professional with 3 Gb RAM. For the PCA, a separate model was built for each tissue; three models in total—one for serum, one for CSF and one for brain tissue. The metabolite intensity data was mean centered and scaled to unit variance and the models were fitted using the autofit function. For the brain samples, PLS-DA model was fitted using the autofit function on mean centered unit variance scaled data.

## 3. Results

### 3.1. Effect of DSP4 Pretreatment on Monoamine Levels in Brain Tissue

**Figure 1:**
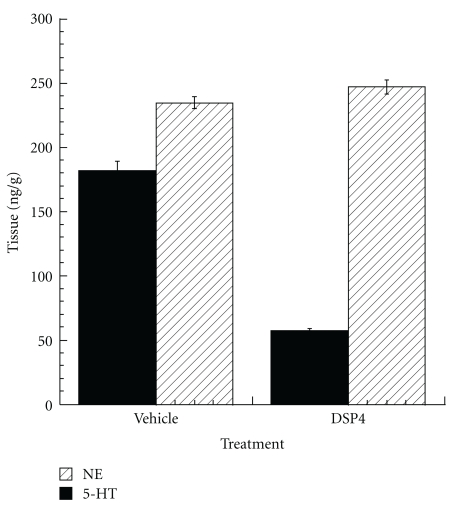
Effects of treatment with DSP4 on monoamine neurotransmitter levels. DSP4 (50 mg/kg in PBS, *N* = 5) or PBS (vehicle, *N* = 5) was administered intraperitoneally to C57BL/6SJL mice on days 1 and 7. Mice were sacrificed 7 days following the second injection (day 14). Brain was harvested and frontal cortex dissected for analysis of monoamine concentrations, demonstrating a 68% reduction of NE levels but no effect on serotonin.

Analyses of neurotransmitters in frontal cortices dissected from C57BL/6SJL mice on day 14, after treatment with DSP4 or vehicle on days 1 and 7, demonstrated a 68% reduction in the levels of norepinephrine, but no detectable effect on levels of serotonin (Figure 1).

### 3.2. Platform QC and Study Precision

The quality control data for this study can be summarized as follows: for plasma samples, standards (*n* = 20) in CMTRX yielded a median relative standard deviation (RSD) of 6.5%, nonstandards (*n* = 326) in CMTRX yielded a median RSD of 17%; for CSF samples, standards (*n* = 20) in CMTRX yielded a median RSD of 6.5%, nonstandards (*n* = 68) in CMTRX yielded a median RSD of 17%; for brain samples, standards (*n* = 20) in CMTRX yielded a median RSD of 11%, nonstandards (*n* = 194) in CMTRX yielded a median RSD of 23%. Results of this analysis indicated that the platform performed well within Metabolon's QC specifications for both plasma and CSF matrices. The brain RSD is higher because of the significant presence of lipids in brain tissue.

### 3.3. Metabolite Summary

Full data curation of the plasma samples yielded 487 chemical entities. 152 of these corresponded to identifiable chemical compounds and the remaining represented currently unnamed compounds. The CSF samples yielded 115 chemical entities, which is a little less than is typically seen due to the smaller sample volume of mouse CSF. 61 of these corresponded to identifiable chemical compounds and the remaining represented unnamed compounds. The brain tissue samples yielded 232 chemical entities. 115 of these corresponded to identifiable chemical compounds, and the remaining represented unnamed compounds. Results for individual metabolites are shown as Whisker plots demarcating upper and lower quartiles and further annotated with mean and median values.

### 3.4. Principal Component Analysis (PCA) and Partial Least Squares-Discriminant Analysis (PLS-DA)

**Figure 2:**
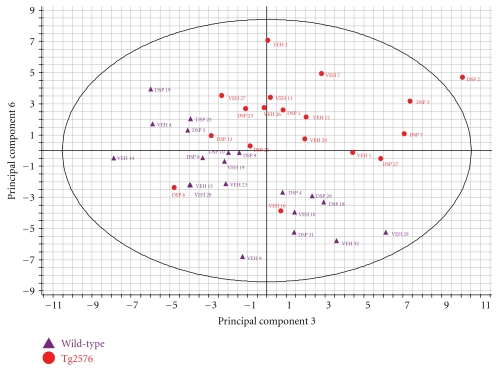
Principal components analysis. From an autofit model that yielded 7 principal components, a combination of PC3 and PC6 gives a separation of the wild type and transgenic samples of brain metabolites. This figure shows the scores plot, derived by principal components analysis, as a combination of Principal Component (PC) 3 on the *x*-axis and PC 6 on the *y*-axis. Each transgenic animal is represented by a red circle, each wild type by a purple triangle. Each point is labeled either with VEH for vehicle treatment or DSP for drug treatment followed by the number assigned to an individual mouse after assignment to a treatment group, but before any experimental procedure. The clustering observed in this plot indicates separation of the wild-type and transgenic samples based on the global profile of all of the brain metabolites detected in our analysis.

For the plasma specimens, autofit function generated a model containing 6 principal components that captured 56% of the total variance. The principal components do not correspond with experimental factors and the experimental groups do not segregate clearly. For the CSF specimens, autofit function generated a model containing 3 principal components that captured 57% of the total variance. The scores plot highlighted mouse Veh 11 as a potential outlier sample. The principal components did not correspond with experimental factors and the experimental groups did not clearly segregate. In contrast, for the brain specimens, autofit generated a model containing 7 principal components that captured 48% of the total variance. The majority of principal components did not correspond to a specific experimental factor but a combination of PC3 and PC6 gave a separation of the wild type and transgenic samples of brain metabolites. This observation was confirmed using PLS-DA, using the wild type and Tg2576 animals as groups. The Variable Importance in Projection (VIP) plot highlighted the carbohydrate and oxidative stress metabolites as the most significant in driving the separation between the wild-type and Tg2576 data sets in the PLS-DA analysis (Figure 2).

### 3.5. Overview of Biochemical Findings

**Figure 3:**
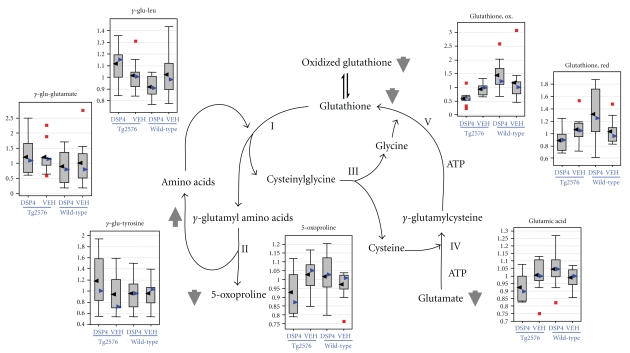
Glutathione metabolism. Glutathione metabolism and gamma-glutamyl-dipeptide formation are altered in the brains of the Tg2576_DSP4 group. For each metabolite, the distribution of values within each of the four cohorts is represented as a Whisker plot, with the relative normalized intensity as the abscissa. Mean values represented by the black arrowheads, median values by blue arrowheads. Detailed statistical comparisons of oxidized glutathione, reduced glutathione, and  *γ*-glutamine-leucine are given in Table 1. Glutamic acid was omitted from the table even though the *P*-value for the comparison of ablated transgenics to ablated wild-types was  .0109, the *Q*-value exceeded our threshold at a value of 0.154.

**Figure 4:**
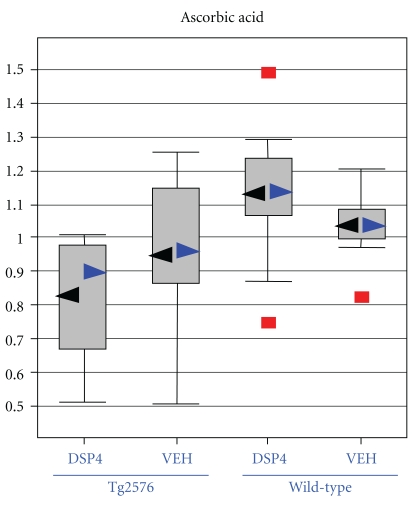
Ascorbic acid, another marker of oxidative stress. Box plots for levels of ascorbic acid in the brain, displaying the distribution of values within each of the four cohorts represented as a Whisker plot, with the relative normalized intensity as the abscissa. Mean values represented by the black arrowheads, median values by blue arrowheads. Detailed statistical comparisons of ascorbate in the four cohorts are given in Table 1.

**Figure 5:**
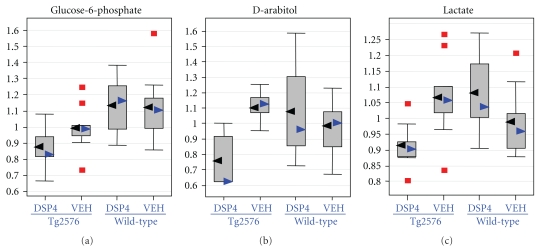
Carbohydrate metabolism. Box plots demonstrating selective depressions in the brains of glucose-6-phosphate (an early intermediary of anaerobic glycolysis as well as the pentose monophosphate shunt), lactate the end product of anaerobic glycolysis, and D-arabitol, a polyol with antioxidant properties which is thought to be a product of the pentose phosphate pathway. For each metabolite, the distribution of values within each of the four cohorts is represented as a Whisker plot, with the relative normalized intensity as the abscissa. Mean values represented by the black arrowheads, median values by blue arrowheads. Detailed statistical comparisons of each of the pictured metabolites in the four cohorts are given in Table 1.

**Figure 6:**
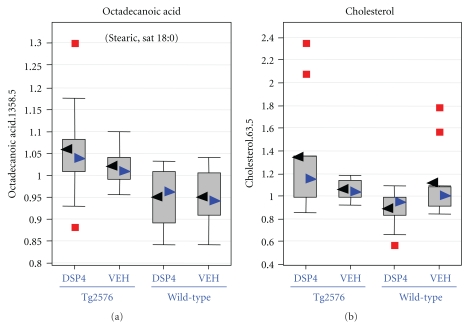
Lipids: Octadecanoic (stearic) acid and cholesterol. Box plots describing the modest elevations of octadecanoic acid and cholesterol in the brains of transgenic animals in which the locus coeruleus had been ablated. For each metabolite, the distribution of values within each of the four cohorts is represented as a Whisker plot, with the relative normalized intensity as the abscissa. Mean values represented by the black arrowheads, median values by blue arrowheads. Detailed statistical comparisons of both of these metabolites in the four cohorts are given in Table 1.

Statistically significant differences (*P* ≤ .05) were detected from amyloid transgenesis alone (i.e., differences between transgenics and the appropriate control group of wild-type animals, as matched with respect to treatment with DSP4 or vehicle), and from LC ablation alone (i.e., differences between DSP4-treated animals and the appropriate control group of vehicle animals, as matched with respect to transgenesis). However, of greatest interest were those changes that required both amyloid transgenesis and also ablation of the LC, that is, those in which Tg2576_DSP4 versus Tg2576_VEH showed statistically significant changes but WT_DSP4 versus WT_VEH did not. We will restrict the analysis in this paper to those changes observed in the brain as a result of LC ablation in Tg2576 transgenic animals, but not observed in wild-type animals, at a more stringent *P*-value of ≤.01 (Table 1). In this latter group the major biochemical findings are: (1) The Tg2576_DSP4 mice already show signs of oxidative stress at 28 weeks of age, as demonstrated by decreased levels of several antioxidants such as glutathione (Figure 3) and increases of oxidative stress metabolite, ascorbate (Figure 4); (2) the Tg2576_DSP4 group shows an overall decrease of carbohydrates in the glycolysis/gluconeogenesis and pentose phosphate pathways, as well as minimal alterations of lactate, which varied between the three tissue compartments (Figure 5). This is consistent with decreased glucose turnover described in the literature on AD; (3) changes in brain lipids, modest increases in cholesterol and octadecanoic (stearic) acid (Figure 6) in transgenics in which the LC was ablated, in comparison to wild-type animals with the same LC ablation.

### 3.6. Comparisons of Specific Biochemical Pathways

#### 3.6.1. Oxidative Stress

Many small molecules involved in the metabolism of glutathione, the major nonenzymatic cellular antioxidant, showed significant changes in the brain tissue of the Tg2576_DSP4 group (Figure 3 and Table 1). Of all the metabolic comparisons performed in this study, the most statistically significant is the decrease of oxidized glutathione in brain tissue of lesioned transgenic mice (Tg2576_DSP4) to that in wild-type mice with the same lesion (wt_DSP4). This comparison is significant at *P*-value of <.0005 and a *Q*-value of 0.006. This appears to depend on both the presence of amyloid and the absence of the LC inasmuch as it is not seen in the comparison of wild-type animals with transgenics in which the LC had been left intact. Comparison of levels of oxidized glutathione in Tg2576_VEH to wt_VEH only yields differences with nonsignificant *P* = .536 and *Q* = 0.697. The same comparison with reduced glutathione in the brain yields a *P* = .005, albeit with a marginal *Q*-value of 0.078. Similarly, this effect also requires both amyloid and the absence of the LC, since there is no difference in reduced glutathione levels between wild-type and transgenic animals in which the LC was left intact (*P* = .829, *Q* = 0.752) (Table 1). 

There were also changes in the Tg2576_DSP4 brain levels of all examined *γ*-glutamyl-peptides, which are produced by *γ*-glutamyl transpeptidase- (GGT-) catalyzed cleavage of  *γ*-glutamyl residues from glutathione and transfer to amino acids [56–59]. Of these, the most significant comparison is that of *γ*-glutamyl-leucine which is increased in Tg2576_DSP4 brains to 1.23-fold the levels seen in wt_DSP4 brains, with *P* = .006 and *Q* = 0.081. As in the case for oxidized and reduced glutathione, comparison of transgenic and wild-type animals does not demonstrate a difference in the presence of an intact LC (Table 1). Similar trends were seen for the other *γ*-glutamyl peptides, albeit not at statistically significant levels (Figure 3). *γ*-glutamyl transpeptidase- (GGT-) catalyzed cleavage plays important roles in glutathione metabolism, amino acid transfer and metabolism of toxic compounds [56–59]. Therefore, the altered levels of *γ*-glutamyl dipeptides in the Tg2576_DSP4 group (Figure 3) are another indication of increased stress conditions in this group.

Ascorbic acid levels (Figure 4) were severely reduced in Tg2576_DSP4 brains (Table 1; *P* = .003, *Q* = 0.051) consistent with reports that levels of ascorbate are altered in AD [17]. As in the case of the glutathione pathway, there is no significant difference in brain ascorbate levels in transgenic and wild-type animals in which the LC remained intact (Table 1). Paradoxically, there was an almost three-fold increase in the uric acid level in the brains of the Tg2576_DSP4 group in comparison to the wt_DSP4 group whereas the amount of uric acid in the transgenic animals was 63% that of wild type when the LC was left intact (Table 1). In summary, the significant changes of antioxidants and oxidative stress markers in the Tg2576_DSP4 group support the model that transgenic Tg2576 mice, injected with DSP4, show increased levels of oxidative stress as is seen in AD.

#### 3.6.2. Energy Metabolism

We observed an overall decrease of carbohydrates involved in glycolysis, gluconeogenesis, and the pentose phosphate pathway in Tg2576_DSP4 mice. The most significant change was a decrease of the brain levels of glucose-6-phosphate in Tg2576_DSP4 brains compared to wt_DSP4 brains (*P* = .001, *Q* = 0.051) (Table 1 and Figure 5), a difference not observed between brains of unlesioned animals. Another possible indication of activation of the pentose phosphate pathway is the equally significant lowering of brain levels of D-arabitol, a naturally occurring polyol with antioxidant properties that is synthesized in the brain [57]. As is the case for the other antioxidant metabolites described in earlier paragraphs, it is exclusively altered in the brains of transgenic mice in whom the LC has been lesioned (Tg2576_DSP4) with *P* = .001, *Q* = 0.051 (Table 1 and Figure 5). In the presence of an intact LC, transgenic mice do not have a detectable difference in brain levels of d-arabitol from wild-type mice. The third significant alteration relating to energy metabolism in the brains of Tg2576_DSP4 mice is a minimal decrease in lactate levels (*P* = .002, *Q* = 0.051) (Table 1 and Figure 5). 

Nearly all metabolites of the tricarboxylic acid (TCA) cycle showed significant changes between wild-type and Tg2576 transgenic mice, and between DSP4 and vehicle mice. The TCA cycle intermediates are significantly increased in Tg2576_vehicle mice compared to wild-type vehicle mice. However, treatment with DSP4 reduced the TCA intermediates in both wild type and transgenic mice backgrounds. Some TCA metabolites show rather minor changes in the wild-type_DSP4 versus wild-type_VEH group, but a significant change in the Tg2576_DSP4 versus Tg2576_VEH comparison. The most statistically significant change was for malic acid levels in the brains of Tg2576_DSP4 which were shown to be minimally altered at 0.89-fold the level in wt_DSP4 brains, at a *P* = .032, *Q* = 0.212. However, there was an even more significant alteration (*P* = .007, *Q* = 0.206) comparing wild type and transgenic mice in whom the LC was intact, albeit in the opposite direction. In summary, the Tg2576_DSP4 mice showed reductions in carbohydrates involved in the glycolysis, gluconeogenesis and pentose phosphate pathways compared to wild type controls.

#### 3.6.3. Free Fatty Acids and Lipids

A diverse array of changes in free fatty acids (FFA) and lipids were observed in this study. However, only the change in octadecanoic (stearic) acid was found to approach statistical significance (*P* = .009, *Q* = 0.101) comparing Tg2576_DSP4 to wt_DSP4 mice (Table 1 and Figure 6). Brain cholesterol showed the same trend. However, several other lipid-derived brain metabolites showed a decrease in the Tg2576_DSP4 group compared to the other treatment groups.

## 4. Discussion

The PCA analysis identified group differences between the metabolic profiles in the brains of wild type and Tg2576 animals. These differences are subtle in that they are not highlighted in the first principal components and the sum of the variance of the principal components is only 11% of the total variance. The PLS-DA analysis allows us to identify the metabolites which contribute most to the separation of the wild type and Tg2576 animal groups. These metabolites are involved in carbohydrate metabolism and oxidative stress response, which corresponds to the metabolites identified as significantly differentially expressed in the univariate analysis of the metabolite data. These changes also correspond with known changes in the brain in AD where, it has been long established that glucose metabolism is impaired in AD [12, 60]. The multivariate analysis of the metabolite data does not reveal much significance over and above the univariate analysis. With the exception of the genotype differences in the brain samples, the other models fail to capture more than 60% of the total variance. The majority of single or pairs of principal components do not correspond to either the drug or genotype experimental factors in the plasma or the CSF. For this reason, we chose to limit this analysis to the brain, even though multiple metabolites in plasma and CSF did show apparently significant differences in individual comparisons.

Mindful of the hazards attendant to multiple comparisons, we chose to focus on those brain metabolites that demonstrated differences with a more restrictive nominal *P*-value of ≤.01 (Table 1). In this set of 10 metabolites, six fell effectively within the conventional cutoff value for False Discovery Rate of *Q* ≤ 0.05 and the remaining four within the range 0.05 < *Q* ≤ 0.10, indicating trends. Eight of these 10 most significantly altered metabolites relate to oxidative stress.

### 4.1. Oxidative Stress

The most significant change was in the levels of glutathione, the major antioxidant protecting cells from toxins such as free radicals. Glutathione is found almost exclusively in its reduced form, since the enzyme which reverts it from its oxidized form is constitutively active and inducible upon oxidative stress. The severe depletion of glutathione and its metabolites in the Tg2576_DSP4 brains is consistent with the presence of increased oxidative stress in these mice. This is an important finding since previous studies examining the relationship between glutathione and AD have been inconclusive [61, 62]. It is of interest that both reduced and oxidized forms of glutathione were lowered, indicating decreased synthesis or increased degradation. Whatever the mechanism, these indicate a decreased capacity to scavenge free radicals. Further support for the significance of the observed differences in the glutathione cycle come from the analysis of concordant trends in the *γ*-glutamyl peptides, of which the alteration of *γ*-glutamyl-leucine approached statistical significance (Table 1).

In addition to changes directly related to the glutathione cycle, there was a statistically significant reduction of ascorbic acid in the brains of transgenic animals in whom the LC was ablated (*P* = .003, *Q* = 0.051) whereas no difference was observed when the LC had been left intact (Table 1). Reduced levels of ascorbate had been observed in AD [17]. Surprisingly, we also observed an apparently discordant result in the elevation of uric acid levels in the brain of Tg2756_DSP4 mice, instead of the expected reduction, which was observed in the unlesioned transgenics. Uric acid is a scavenger of ROS, about as effective as vitamin C. Tohgi et al. [63] found that uric acid and xanthine are much lower in AD CSF presumably because of impaired brain metabolism. The increase of uric acid in our experimental animals appears robust but unexplained. Similarly, there were equivocal differences seen in levels of tocopherol. Kontush and Schekatolina [64] have shown that vitamin E levels in AD subjects vary widely depending on the study. Furthermore, it is possible that some antioxidants show significant changes in this study while others do not show the same extent of changes because of only partial depletion of norepinephrine (63%) or the relatively young age (23 weeks) of the transgenic mice used in this study. Kawarabayashi et al. [65] have previously shown that the accumulation of amyloid *β* plaques in the Tg2576 mouse model only reach the levels seen in AD brain between 15 and 23 months of age.

### 4.2. Energy Metabolism

Less direct but nevertheless compelling evidence of increased oxidative stress in the brains of Tg2756_DSP4 animals derives from our observations of significantly altered levels of glucose-6-phosphate and of the polyol D-arabitol (Table 1) [66–69]. Glucose-6-phosphate can undergo a number of metabolic transformations. Besides being an intermediate in the Embden-Meyerhof pathway, where it is reversibly isomerized to D-fructose-6-phosphate by phosphoglucose isomerase, it can also be irreversibly metabolized by NADP-linked glucose-6-phosphate dehydrogenase in the first step of the oxidative pentose phosphate pathway, or by phosphoglucomutase in the first step of the glucuronic acid pathway [66]. Glucose-6-phosphate dehydrogenase plays a pivotal role in homeostatic redox control by providing reducing equivalents to glutathione. Thus, lower levels of glucose-6-phosphate in the brains of transgenic animals in which the LC has been ablated could indicate either decreased glucose uptake or increased flux through the pentose monophosphate shunt, which has been shown to be induced in AD [67], presumably to provide reductive compensation to oxidative stress. Furthermore, the striking alteration in the levels of D-arabitol, a little-studied polyol that is believed to be derived from sugar phosphates in the pentose monophosphate pathway, further supports alteration of this pathway. However, the metabolic pathways for these and other brain derived polyols have not yet been fully determined [68], so that our interpretation must be considered speculative.

There is extensive literature documenting altered metabolism of carbohydrates in AD [12, 60, 70]. Our claim of greater concordance of LC-lesioned transgenic mice with human AD is consistent with the general consensus in the literature that glucose and carbohydrate metabolism, and therefore the formation of cellular energy is reduced severely in AD. Bigl et al. [71] observed a lowered glucose metabolism in Tg2576 mice as well, but only at 24 months of age were reductions in the key enzymes in the glycolysis and gluconeogenesis pathway detected.

Several metabolites in the TCA cycle demonstrated an interaction effect between the genotype and drug (data not shown), most notably malate. However, malate was also significantly altered in comparisons of transgenics and wild type animals in which the LC had been left intact. TCA cycle proteins prone to reactive oxygen species (ROS) damage include aconitase, pyruvate dehydrogenase and alpha-ketoglutarate dehydrogenase, all of which have been shown to be significantly reduced in AD [72–75].

### 4.3. Free Fatty Acids and Lipids

The remaining two of the ten most statistically significant contrasts in the Tg2756_DSP4 brains relate to lipid metabolism, neither of which can we relate readily to oxidative stress. There is a statistically significant elevation in cholesterol in lesioned transgenic animals, but no difference from wild-type in transgenic animals with an intact LC (Table 1). The relationship of cholesterol to autopsy findings in AD is complex, depending on one's use of the historical or the currently accepted definition of AD. Up to this point in our discussion, we have used the term AD exclusively to refer to sporadic, senile onset Alzheimer's disease, as it is referred to in the current literature, but which had previously been referred to as SDAT, Senile Dementia of the Alzheimer Type. This distinction is germane to considerations of cholesterol metabolism. Two independent autopsy studies have demonstrated a decrease in cholesterol in the cerebral white matter in autopsies of patients with Alzheimer's disease as defined historically—that is, autosomal dominant presenile dementia as described by Alois Alzheimer [76]—in comparison with age-matched controls or patients with SDAT. In contrast, cholesterol and certain other lipids were reduced in the frontal centrum semiovale of patients with SDAT in comparison to age-matched controls or patients with presenile dementia—referred to by the authors of that report as “true Alzheimer's disease” [77]. It is of further interest that cholesterol loading was found to modulate A*β* toxicity through depletion of mitochondrial stores of glutathione, possibly furnishing an indirect link to oxidative stress [78]. Be that as it may, we cannot offer a meaningful interpretation of this statistically significant difference in cholesterol. Similarly, we do not understand the modest elevation of octadecanoic (stearic) acid in the brains of Tg2756_DSP4 mice, but note its concordance with observations in autopsy studies of Alzheimer's disease [79] as well as to a report of its ability to induce hyperphosphorylation of tau in an experimental system [80].

## 5. Conclusion

This data provides further support to the original hypothesis by Heneka et al. [42, 43], that loss of the widely projecting noradrenergic LC is not just an incidental finding but may contribute to key pathophysiological features of human Alzheimer's disease. Furthermore, it extends this hypothesis from inflammation to oxidative stress and altered carbohydrate metabolism. It is an interesting historical note that after an extensive body of work on the role of the noradrenergic LC in Alzheimer's disease [30–41, 81], most investigators shifted their focus to the cholinergic substantia innominata, in which cell loss is not as extensive [40]. Perhaps this deserves reappraisal.

If the importance of the loss of the LC is corroborated by studies of other markers of oxidative damage, such as 8-hydroxyguanosine [82], 4-hydroxy-2-nonenal (HNE), isoprostanes [83] and nitrotyrosine [84], this may have practical consequences in the development of animal models more congruent with the human disease. An important test of this extended hypothesis will be comparison of cell loss and cognitive function in amyloid transgenics in which the LC has been ablated. If confirmed, not only would this have practical consequences in the design of animal models, but would also provide an experimental test of the hypothesis that free radicals are the rate limiting step in this as well as other neurodegenerative disorders [22].

## Figures and Tables

**Table 1:** The 10 most statistically significant metabolic differences in brain tissue, associated with transgenesis for Tg2576 that were observed after ablation of the locus coeruleus with DSP4 but not in animals in which the locus coeruleus had been left intact. The *P*-value describes the false positive rate, the *Q*-value the false discovery rate.

Compound	Locus coeruleus ablated: Tg/wt		Locus coeruleus intact: Tg/wt		Tg-ablated/wt-ablated (Ratio of means)	Tg-intact/wt-intact (Ratio of means)
	*P*	*Q*	*P*	*Q*		
Glutathione, oxidized	.000	0.006	.536	0.697	0.41	0.80
Glucose-6-phosphate	.001	0.051	.121	0.458	0.78	0.88
D-arabitol	.001	0.051	.193	0.505	0.69	1.12
Cholesterol	.002	0.051	.853	0.752	1.51	0.95
Lactate	.002	0.051	.135	0.461	0.84	1.06
Ascorbic acid	.003	0.051	.251	0.530	0.73	0.92
Glutathione, reduced	.005	0.078	.829	0.752	0.68	1.03
*γ*-glutamyl-leucine	.006	0.081	.968	0.777	1.23	0.99
Octadecanoic acid	.009	0.101	.058	0.423	1.12	1.07
Uric acid	.010	0.101	.576	0.715	2.91	0.63

## References

[1] Hsiao K, Chapman P, Nilsen S (1996). Correlative memory deficits, A*β* elevation, and amyloid plaques in transgenic mice. *Science*.

[2] Cai X-D, Golde TE, Younkin SG (1993). Release of excess amyloid *β* protein from a mutant amyloid *β* protein precursor. *Science*.

[3] Cai X-D, Golde TE, Younkin SG (1993). Release of excess amyloid *β* protein from a mutant amyloid *β* protein precursor. *Science*.

[4] Hsiao K, Chapman P, Nilsen S (1996). Correlative memory deficits, A*β* elevation, and amyloid plaques in transgenic mice. *Science*.

[5] Riley KP, Snowdon DA, Desrosiers MF, Markesbery WR (2005). Early life linguistic ability, late life cognitive function, and neuropathology: findings from the Nun Study. *Neurobiology of Aging*.

[6] Iacono D, Markesbery WR, Gross M (2009). The nun study: clinically silent AD, neuronal hypertrophy, and linguistic skills in early life. *Neurology*.

[7] Fernando MS, Ince PG (2004). Vascular pathologies and cognition in a population-based cohort of elderly people. *Journal of the Neurological Sciences*.

[8] Andersen JK (2004). Oxidative stress in neurodegeneration: cause or consequence?. *Nature Medicine*.

[9] Baldeiras I, Santana I, Proença MT (2008). Peripheral oxidative damage in mild cognitive impairment and mild Alzheimer’s disease. *Journal of Alzheimer’s Disease*.

[10] De La Monte SM, Wands JR (2006). Molecular indices of oxidative stress and mitochondrial dysfunction occur early and often progress with severity of Alzheimer’s disease. *Journal of Alzheimer’s Disease*.

[11] Keller JN (2006). Interplay between oxidative damage, protein synthesis, and protein degradation in Alzheimer’s disease. *Journal of Biomedicine and Biotechnology*.

[12] Hoyer S (1996). Oxidative metabolism deficiencies in brains of patients with Alzheimer’s disease. *Acta Neurologica Scandinavica, Supplement*.

[13] Kim T-S, Pae C-U, Yoon S-J (2006). Decreased plasma antioxidants in patients with Alzheimer’s disease. *International Journal of Geriatric Psychiatry*.

[14] Lovell MA, Markesbery WR (2007). Oxidative DNA damage in mild cognitive impairment and late-stage Alzheimer’s disease. *Nucleic Acids Research*.

[15] Lovell MA, Markesbery WR (2007). Oxidative damage in mild cognitive impairment and early Alzheimer’s disease. *Journal of Neuroscience Research*.

[16] Markesbery WR, Lovell MA (2007). Damage to lipids, proteins, DNA, and RNA in mild cognitive impairment. *Archives of Neurology*.

[17] Mecocci P (2004). Oxidative stress in mild cognitive impairment and Alzheimer disease: a continuum. *Journal of Alzheimer’s Disease*.

[18] Mosconi L, Pupi A, De Leon MJ (2008). Brain glucose hypometabolism and oxidative stress in preclinical Alzheimer’s disease. *Annals of the New York Academy of Sciences*.

[19] Petersen RB, Nunomura A, Lee H-G (2007). Signal transduction cascades associated with oxidative stress in Alzheimer’s disease. *Journal of Alzheimer’s Disease*.

[20] Schulz JB, Lindenau J, Seyfried J, Dichgans J (2000). Glutathione, oxidative stress and neurodegeneration. *European Journal of Biochemistry*.

[21] Su B, Wang X, Nunomura A (2008). Oxidative stress signaling in Alzheimer’s disease. *Current Alzheimer Research*.

[22] Wright AF, Jacobson SG, Cideciyan AV (2004). Lifespan and mitochondrial control of neurodegeneration. *Nature Genetics*.

[23] Akiyama H, Barger S, Barnum S (2000). Inflammation and Alzheimer’s disease. *Neurobiology of Aging*.

[24] Yao Y, Chinnici C, Tang H, Trojanowski JQ, Lee VMY, Praticò D (2004). Brain inflammation and oxidative stress in a transgenic mouse model of Alzheimer-like brain amyloidosis. *Journal of Neuroinflammation*.

[25] Karuppagounder SS, Xu H, Shi Q (2009). Thiamine deficiency induces oxidative stress and exacerbates the plaque pathology in Alzheimer’s mouse model. *Neurobiology of Aging*.

[26] Resende R, Moreira PI, Proença T (2008). Brain oxidative stress in a triple-transgenic mouse model of Alzheimer disease. *Free Radical Biology and Medicine*.

[27] Lee K-W, Kim J-B, Seo J-S (2009). Behavioral stress accelerates plaque pathogenesis in the brain of Tg2576 mice via generation of metabolic oxidative stress. *Journal of Neurochemistry*.

[28] Dumont M, Wille E, Stack C, Calingasan NY, Beal MF, Lin MT (2009). Reduction of oxidative stress, amyloid deposition, and memory deficit by manganese superoxide dismutase overexpression in a transgenic mouse model of Alzheimer’s disease. *The FASEB Journal*.

[29] Butterfield DA, Galvan V, Lange MB (2009). In vivo oxidative stress in brain of Alzheimer disease transgenic mice: requirement for methionine 35 in amyloid *β*-peptide of APP. *Free Radical Biology and Medicine*.

[30] Mann DMA, Lincoln J, Yates PO (1980). Changes in the monoamine containing neurones of the human CNS in senile dementia. *British Journal of Psychiatry*.

[31] Tomlinson BE, Irving D, Blessed G (1981). Cell loss in the locus coeruleus in senile dementia of Alzheimer type. *Journal of the Neurological Sciences*.

[32] Mann DMA, Yates PO, Hawkes J (1983). The pathology of the human locus ceruleus. *Clinical Neuropathology*.

[33] Mann DMA, Yates PO, Marcyniuk B (1984). A comparison of changes in the nucleus basalis and locus caeruleus in Alzheimer’s disease. *Journal of Neurology Neurosurgery and Psychiatry*.

[34] Bondareff W, Mountjoy CQ, Roth M (1982). Loss of neurons of origin of the adrenergic projection to cerebral cortex (nucleus locus ceruleus) in senile dementia. *Neurology*.

[35] Ichimiya Y, Arai H, Kosaka K, Iizuka R (1986). Morphological and biochemical changes in the cholinergic and monoaminergic systems in Alzheimer-type dementia. *Acta Neuropathologica*.

[36] Chan-Palay V, Asan E (1989). Alterations in catecholamine neurons of the locus coeruleus in senile dementia of the Alzheimer type and Parkinson’s disease with and without dementia and depression. *Journal of Comparative Neurology*.

[37] Chan-Palay V (1991). Alterations in the locus coeruleus in dementias of Alzheimer’s and Parkinson’s disease. *Progress in Brain Research*.

[38] German DC, Manaye KF, White CL (1992). Disease-specific patterns of locus coeruleus cell loss. *Annals of Neurology*.

[39] Nazarali AJ, Reynolds GP (1992). Monoamine neurotransmitters and their metabolites in brain regions in Alzheimer’s disease: a postmortem study. *Cellular and Molecular Neurobiology*.

[40] Zarow C, Lyness SA, Mortimer JA, Chui HC (2003). Neuronal loss is greater in the locus coeruleus than nucleus basalis and substantia nigra in Alzheimer and Parkinson diseases. *Archives of Neurology*.

[41] Adolfsson R, Gottfries CG, Roos BE, Winblad B (1979). Changes in the brain catecholamines in patients with dementia of Alzheimer type. *British Journal of Psychiatry*.

[42] Heneka MT, Galea E, Gavriluyk V (2002). Noradrenergic depletion potentiates *β*-amyloid-induced cortical inflammation: implications for Alzheimer’s disease. *Journal of Neuroscience*.

[43] Feinstein DL, Heneka MT, Gavrilyuk V, Dello Russo C, Weinberg G, Galea E (2002). Noradrenergic regulation of inflammatory gene expression in brain. *Neurochemistry International*.

[44] Heneka MT, Gavrilyuk V, Landreth GE, O’Banion MK, Weinberg G, Feinstein DL (2003). Noradrenergic depletion increases inflammatory responses in brain: effects on I*κ*B and HSP70 expression. *Journal of Neurochemistry*.

[45] Heneka MT, Ramanathan M, Jacobs AH (2006). Locus ceruleus degeneration promotes Alzheimer pathogenesis in amyloid precursor protein 23 transgenic mice. *Journal of Neuroscience*.

[46] Kalaria RN, Stockmeier CA, Harik SI (1989). Brain microvessels are innervated by locus ceruleus noradrenergic neurons. *Neuroscience Letters*.

[47] Jonnson G, Hallman H, Fonzio F, Ross S (1981). DSP4 (N-(2-chloroethyl)-N-(ethyl-2-bromobenzylamine)—a useful dennervation tool for central and peripheral noradrenalin neurons. *European Journal of Pharmacology*.

[48] Landa ME, Rubio MC, Jaim-Etcheverry G (1984). The neurotoxic compound N-(2-chloroethyl)-N-ethyl-2-bromobenzylamine hydrochloride (DSP4) depletes endogenous norepinephrine and enhances release of [*3H*]norepinephrine from rat cortical slices. *Journal of Pharmacology and Experimental Therapeutics*.

[49] Boudonck KJ, Mitchell MW, Német L (2009). Discovery of metabolomics biomarkers for early detection of nephrotoxicity. *Toxicologic Pathology*.

[50] Boudonck KJ, Rose DJ, Karoly ED, Lee DP, Lawton KA, Lapinskas PJ (2009). Metabolomics for early detection of drug-induced kidney injury: review of current status. *Bioanalysis*.

[51] Evans AM, DeHaven CD, Barrett T, Mitchell M, Milgram E (2009). Integrated, nontargeted ultrahigh performance liquid chromatography/electrospray ionization tandem mass spectrometry platform for the identification and relative quantification of the small-molecule complement of biological systems. *Analytical Chemistry*.

[52] Benjamini Y, Hochberg Y (1995). Controlling the false discovery rate: a practical and powerful approach to multiple testing. *Journal of the Royal Statistical Society: Series B*.

[53] Storey JD, Tibshirani R (2003). Statistical significance for genomewide studies. *Proceedings of the National Academy of Sciences of the United States of America*.

[54] Pearson K (1901). On lines and planes of closest fit to systems of points in space. *Philosophical Magazine*.

[55] Gottfries J, Sjögren M, Holmberg B, Rosengren L, Davidsson P, Blennow K (2004). Proteomics for drug target discovery. *Chemometrics and Intelligent Laboratory Systems*.

[56] Palekar AG, Tate SS, Meister A (1974). Formation of 5 oxoproline from glutathione in erythrocytes by the *γ* glutamyltranspeptidase cyclotransferase pathway. *Proceedings of the National Academy of Sciences of the United States of America*.

[57] Wang W, Ballatori N (1998). Endogenous glutathione conjugates: occurrence and biological functions. *Pharmacological Reviews*.

[58] Visvikis A, Thioudellet C, Oster T, Fournel-Gigleux S, Wellman M, Siest G (1991). High-level expression of enzymatically active mature human *γ*-glutamyltransferase in transgenic V79 Chinese hamster cells. *Proceedings of the National Academy of Sciences of the United States of America*.

[59] Lieberman MW, Wiseman AL, Shi Z-Z (1996). Growth retardation and cysteine deficiency in *γ*-glutamyl transpeptidase-deficient mice. *Proceedings of the National Academy of Sciences of the United States of America*.

[60] Lying-Tunell U, Lindblad BS, Malmlund HO, Persson B (1981). Cerebral blood flow and metabolic rate of oxygen, glucose, lactate, pyruvate, ketone bodies and amino acids. II. Presenile dementia and normal-pressure hydrocephalus. *Acta Neurologica Scandinavica*.

[61] Schulz JB, Lindenau J, Seyfried J, Dichgans J (2000). Glutathione, oxidative stress and neurodegeneration. *European Journal of Biochemistry*.

[62] Vitvitsky V, Thomas M, Ghorpade A, Gendelman HE, Banerjee R (2006). A functional transsulfuration pathway in the brain links to glutathione homeostasis. *Journal of Biological Chemistry*.

[63] Tohgi H, Abe T, Takahashi S, Kikuchi T (1993). The urate and xanthine concentrations in the cerebrospinal fluid in patients with vascular dementia of the Binswanger type, Alzheimer type dementia, and Parkinson’s disease. *Journal of Neural Transmission: Parkinson’s Disease and Dementia Section*.

[64] Kontush A, Schekatolina S (2004). Vitamin E in neurodegenerative disorders: Alzheimer’s disease. *Annals of the New York Academy of Sciences*.

[65] Kawarabayashi T, Younkin LH, Saido TC, Shoji M, Ashe KH, Younkin SG (2001). Age-dependent changes in brain, CSF, and plasma amyloid *β* protein in the Tg2576 transgenic mouse model of Alzheimer’s disease. *Journal of Neuroscience*.

[66] Wamelink MMC, Struys EA, Jakobs C (2008). The biochemistry, metabolism and inherited defects of the pentose phosphate pathway: a review. *Journal of Inherited Metabolic Disease*.

[67] Russell RL, Siedlak SL, Raina AK, Bautista JM, Smith MA, Perry G (1999). Increased neuronal glucose-6-phosphate dehydrogenase and sulfhydryl levels indicate reductive compensation to oxidative stress in Alzheimer disease. *Archives of Biochemistry and Biophysics*.

[68] Kusmierz J, DeGeorge JJ, Sweeney D, May C, Rapoport SI (1989). Quantitative analysis of polyols in human plasma and cerebrospinal fluid. *Journal of Chromatography: Biomedical Applications*.

[69] Palmer AM (1999). The activity of the pentose phosphate pathway is increased in response to oxidative stress in Alzheimer’s disease. *Journal of Neural Transmission*.

[70] Meier-Ruge W, Bertoni-Freddari C (1996). The significance of glucose turnover in the brain in the pathogenetic mechanisms of Alzheimer’s disease. *Reviews in the Neurosciences*.

[71] Bigl M, Apelt J, Eschrich K, Schliebs R (2003). Cortical glucose metabolism is altered in aged transgenic Tg2576 mice that demonstrate Alzheimer plaque pathology. *Journal of Neural Transmission*.

[72] Mastrogiacomo F, Bergeron C, Kish SJ (1993). Brain *α*-ketoglutarate dehydrogenase complex activity in Alzheimer’s disease. *Journal of Neurochemistry*.

[73] Gibson GE, Blass JP, Beal MF, Bunik V (2005). The *α*-ketoglutarate-dehydrogenase complex: a mediator between mitochondria and oxidative stress in neurodegeneration. *Molecular Neurobiology*.

[74] Gibson GE, Zhang H, Sheu KF-R (1998). *α*-ketoglutarate dehydrogenase in Alzheimer brains bearing the APP670/671 mutation. *Annals of Neurology*.

[75] Bubber P, Haroutunian V, Fisch G, Blass JP, Gibson GE (2005). Mitochondrial abnormalities in Alzheimer brain: mechanistic implications. *Annals of Neurology*.

[76] Wallin A, Gottfries CG, Karlsson I, Svennerholm L (1989). Decreased myelin lipids in Alzheimer’s disease and vascular dementia. *Acta Neurologica Scandinavica*.

[77] Gottfries C-G, Karlsson I, Svennerholm L (1996). Membrane components separate early-onset Alzheimer’s disease from senile dementia of the Alzheimer type. *International Psychogeriatrics*.

[78] Fernández A, Llacuna L, Fernández-Checa JC, Colell A (2009). Mitochondrial cholesterol loading exacerbates amyloid *β* peptide-induced inflammation and neurotoxicity. *Journal of Neuroscience*.

[79] Prasad MR, Lovell MA, Yatin M, Dhillon H, Markesbery WR (1998). Regional membrane phospholipid alterations in Alzheimer’s disease. *Neurochemical Research*.

[80] Patil S, Chan C (2005). Palmitic and stearic fatty acids induce Alzheimer-like hyperphosphorylation of tau in primary rat cortical neurons. *Neuroscience Letters*.

[81] Braak H, Braak E (1991). Neuropathological stageing of Alzheimer-related changes. *Acta Neuropathologica*.

[82] Moreira PI, Sayre LM, Zhu X, Nunomura A, Smith MA, Perry G (2010). Detection and localization of markers of oxidative stress by in situ methods: application in the study of Alzheimer disease. *Methods in Molecular Biology*.

[83] Irizarry MC, Yao Y, Hyman BT, Growdon JH, Praticò D (2007). Plasma F2A isoprostane levels in Alzheimer’s and Parkinson’s disease. *Neurodegenerative Diseases*.

[84] Butterfield DA, Reed TT, Perluigi M (2007). Elevated levels of 3-nitrotyrosine in brain from subjects with amnestic mild cognitive impairment: implications for the role of nitration in the progression of Alzheimer’s disease. *Brain Research*.

